# Deep Drawing Behavior of Metal-Composite Sandwich Plates

**DOI:** 10.3390/ma15196612

**Published:** 2022-09-23

**Authors:** Shun-Fa Hwang, Yu-Ren Li

**Affiliations:** Department of Mechanical Engineering, National Yunlin University of Science and Technology, Yunlin 64002, Taiwan

**Keywords:** deep drawing, composite, sandwich plate, fiber-metal laminate, blank-holder pressure, fracture, wrinkle

## Abstract

The deep drawing behavior of metal-composite sandwich plates, or fiber-metal laminates, in which aluminum or steel sheets are used as the face layer and composite materials are used as the core layer, is discussed in this work to find the workpieces without fractures and wrinkles. Two types of aluminum, 1050 and 6061, are considered their suitability as face sheets. The preheat effect of aluminum 6061 on formability is also investigated. When steel sheets are used, the effect of blank-holder pressure is included. In addition, to understand the deformation of fabric composite, pure composite laminates are deep drawn. The results of composite laminates show that after drawing, the weft and warp lines are shorter than the other radial lines, causing the specific deformed shape and the variation of the fiber intersection angle. For Al-composite sandwich plates, fractures and wrinkles are easy to occur. Even though the fracture and wrinkle conditions are released with the increase in preheating temperature of aluminum 6061, it may be not enough. For the deep drawing of the steel-composite sandwich plates, in which fractures are avoided, the increase of blank-holder pressure could reduce the wrinkle number. Hence, good quality workpieces without fracture and wrinkle could be obtained when the blank-holder pressure is high enough.

## 1. Introduction

The development of lightweight materials has increased in response to environmental demand. Especially in the automotive industry, lightweight car parts could improve driver safety, fuel efficiency, and environmental contamination. For these reasons, the development of next-generation materials, such as high-strength aluminum alloys, magnesium, and composite materials, attracts a lot of attention [1]. For example, vehicle parts manufactured from advanced high-strength steel have been under development [2]. Being another candidate, composite materials have the advantages of high specific strength and stiffness, non-corrosion, and good fatigue properties. However, there are some limitations, such as low fracture toughness during crashes, production cost, and joining with metals.

One possible alternative is the combination of metals with composite materials into a multi-material system. Metal-composite sandwich plates comprising the metal face layer and the composite core layer are also named fiber-metal laminates (FMLs). One famous application was the use of GLARE (Glass Laminate Aluminum-Reinforced Epoxy) in the upper fuselage panels of the Airbus A380 [3]. Even though the strength of FMLs just falls between the strength of their constituent materials, some properties, like impact performance, are better than the plain constituent material [4]. Furthermore, higher compression strength and damage tolerance of FMLs were indicated [3]. There are a lot of manufacturing methods available for metal-composite structures [5]. Due to the metal face layer, an automated and large-scale production method such as stamp forming could be applied to this metal-composite sandwich plate. To have a complicated shape for vehicle parts, deep drawing that is popular for thin sheet metals [6,7,8] is sometimes necessary. In this method, the curing process of the thermoset composite layer follows stamp forming.

Gresham et al. [9] discussed the drawing behavior of metal-composite sandwich panels with three grades of aluminum alloy as the face layer and two types of reinforced polypropylene composite as the core layer, and the process variables including blank preheat temperature and blank-holder force were investigated. Their results indicated that the blank-holder force has a dominant effect on the failure mode, and the increase in preheat temperature could reduce the failure while increasing the wrinkling severity. Heggemann and Homberg [10] demonstrated that the shape and dimensional accuracy of deep drawn fiber metal laminates could be significantly improved by implementing pre-curing processes before the actual combined forming and curing process. Schmidt et al. [11] optimized the combined forming of sheet metal and composite prepregs based on the detailed knowledge of the material behavior. The application of blank-holder force and blank-holder gap was found to have a positive effect on the formability of GLARE [12]. Blala et al. [13] investigated the influence of the hydroforming process parameters, including cavity pressure, blank-holder force, die entrance radius, blank-holder gap, and punch speed, on FML hemispherical dome made of aluminum 2024-T3 and glass fiber prepreg.

Instead of three-layer sandwiches, a two-layer steel/composite laminate with bonding materials was discussed by Lee et al. [1] for the effect of blank-holder force and the punch velocity. The square cup deep drawing test of this laminate under various process parameters was also conducted by Lee et al. [14] to evaluate the formability. Rajabi et al. [15] and Wollmann et al. [16] replaced the thermoset composite core layer with thermoplastic composite materials for a three-layer sandwich. In addition, it seemed popular to use polymer as the core layer to create metal/polymer/metal sandwiches. Due to their high deformation potential, the formability was investigated for metal/polymer/metal sandwiches [17,18,19,20] and steel/polymer/steel systems with local inlays [21,22]. Among them, the effect of the face/core layer thickness on the deep drawing of sandwiches was investigated [17,18]. Sokolova et al. [23] studied the effect of sample size and core thickness for 316L/PP-PE/316L sandwiches.

Even though the deformation potential of metal/composite/metal sandwiches is less than metal/polymer/metal sandwiches, the former has better specific strength and stiffness and design flexibility for different loading applications. Therefore, it is important to investigate the deep drawing potential of metal/composite/metal sandwiches in response to their potential applications. From the literature, only a few papers were focused on this topic, and among them, the sandwich was drawn to failure to discuss its depth at failure or draw percentage [9]. In this way, both the draw percentage and the blank-holder pressure may be too low to be for real use, and the main part of the sandwich was subjected to just one-side pressure from the punch. Currently, it lacks how to make a sandwich structure with complicated shapes and without failures and wrinkles. Therefore, the present work tries to fulfill these goals by deep drawing with a matched mold creating two-side pressure for the main part of the sandwich. Carbon fiber fabric/epoxy composites are used as the core layer, and aluminum or steel is considered as the metal face sheet. When aluminum 6061 is used as face layers, the preheat effect of aluminum on the formability is investigated. When steel is used as face layers, the blank-holder pressure is increased to obtain workpieces without failures and wrinkles. In addition, to understand the deformation behavior of fabric composites, pure composite laminates are deep drawn.

## 2. Materials and Methods

**Figure 1:**
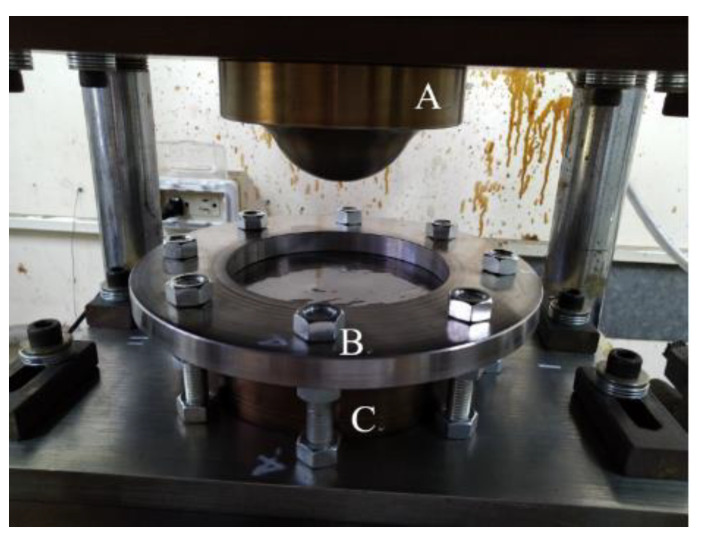
The punch (A), blank-holder (B), and die (C).

**Figure 2:**
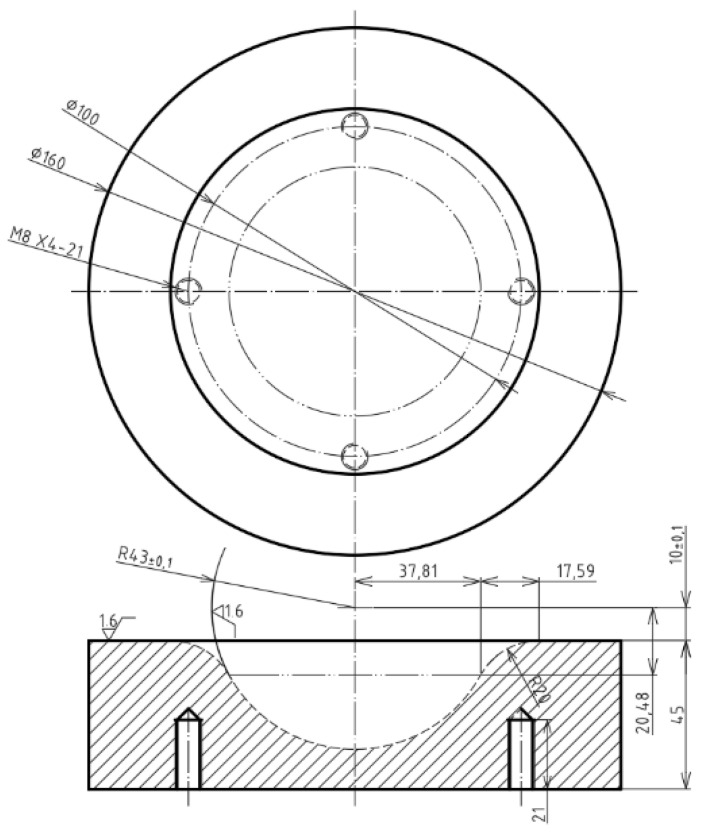
The detailed dimension of the die (unit: mm).

To punch the composite laminate or the sandwich plate, the punch, die, and blank-holder are arranged as shown in Figure 1. The blank was a circular plate with a diameter of 160 mm, and the punch speed was set to be 1.5 mm/s. The blank-holder was a circular plate with an inner diameter of 127 mm and an outer diameter of 225 mm. The applied blank-holder pressure was 0.6 MPa, which is equivalent to a 16.2-kN force. The punch was a hemispherical dome with a radius of 40 mm, while the die as shown in Figure 2 was a 43-mm-radius hemispherical cup. There was a gap of 3 mm between the dome and the cup. In addition, there was a transition zone of 17.59-mm-length from the hemisphere to the flange area, and the radius of this zone was 20 mm. It should be noted that the present deep drawing is a process of matched molds.

To understand the deformation behavior of fabric composites, the 3-mm-thickness composite laminate blank consisting of 11 layers of carbon fiber fabric/epoxy prepregs was deep drawn first. The fiber fabric was plain woven of 8-µm-diameter TC-36S 3K carbon roving that was made by Formosa Plastic Corporation. The Young’s modulus and ultimate strength of the composite material were about 70 GPa and 750 MPa, respectively, as shown in Table 1. Since the fabric was plain woven, the properties of the composite along weft and warp directions were approximately the same. There are four types of composite specimens as listed in Table 2. Each parenthesis represents one woven composite layer with the indicated weft and warp fiber angles with respect to a reference coordinate. Since they are woven composite layers, (+60/−30) layer could be obtained by rotating the (0/90) layer. The specimen of composite-15, in which the weft fiber just has an angle difference of 15° between two consecutive layers, has a more even fiber distribution than the composite-90 specimen. After being deep drawn at room temperature and with the blank-holder pressure of 0.6 MPa, these composite blanks were heated to 140 °C for 30 min in the matched molds and kept at this temperature for 40 min. The heat sources were embedded in the punch and the die, and the blank was heated by conduction. There was no temperature control on the blank-holder. The workpiece with a thickness of 3 mm was demolded after it was reduced to room temperature. The temperature of the blank was measured by a thermocouple.

**Figure 3:**
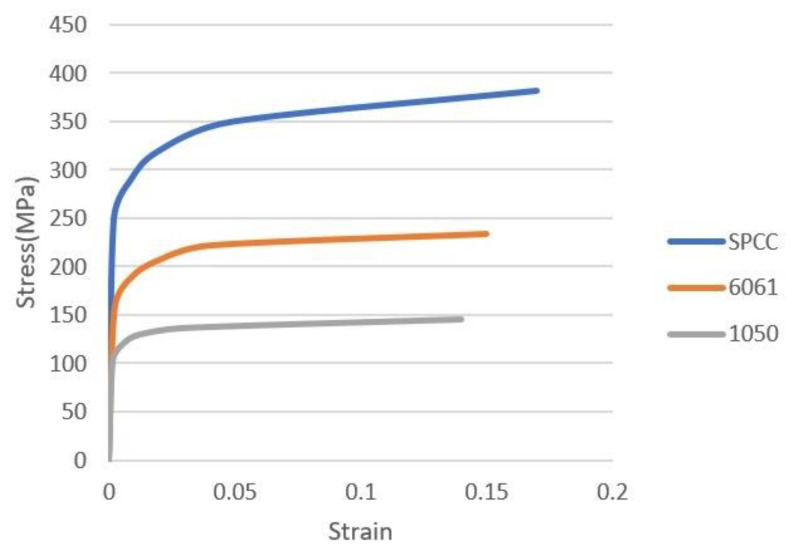
Stress-strain curves of Al 1050, Al 6061, and SPCC steel.

The first type of metal-composite sandwich used aluminum with a thickness of 0.5 mm as the face sheet and the same composite material with [(0/90)]_7_ as the core layer having 2-mm-thickness. Two types of aluminum, 1050 and 6061, were selected because of their availability. Their mechanical properties are listed in Table 1 and the stress-strain curves are shown in Figure 3. The total thickness of the sandwich was 3 mm. By applying Bechem lubricant between the metal and the mold, these two types of Al-composite sandwiches were deep drawn at room temperature with the blank-holder pressure of 0.6 MPa. After that, the same curing process as that for the pure composite blank was executed. In addition, a two-stage drawing process was proposed for Al 6061-composite sandwiches. In the first stage, the pure aluminum, die, and punch was preheated to 140, 190, or 230 °C and deep drawn to reduce the possible fracture. In the second stage, two pieces of the drawn aluminum were combined with the punch and the die, and then the core composite layer at room temperature was drawn with the blank-holder pressure of 0.6 MPa. After that, the curing process as that for the pure composite was executed.

The second type of metal-composite sandwich used the same composite laminate with [(0/90)]_7_ as the core layer. The face sheet material was SPCC steel, a commercial quality cold rolled steel defined in JIS G 3141 standard. This steel is primarily used in automobile parts manufacturing, and its mechanical properties and stress-strain curve are shown in Table 1 and Figure 3, respectively. The original drawing process without preheating was executed. In this part, the pressure from the blank-holder varied from 0.6, 1.1, 2.5, 3.5, 4.0, to 5.5 MPa by controlling the tension force of 8 bolts that were even distributed in the holder. By multiplying the area of the blank-holder, the corresponding blank-holder forces are 16.2, 29.7, 67.5, 94.5, 108.0, and 148.5 kN.

## 3. Results

### 3.1. Composite Plates

**Figure 4:**
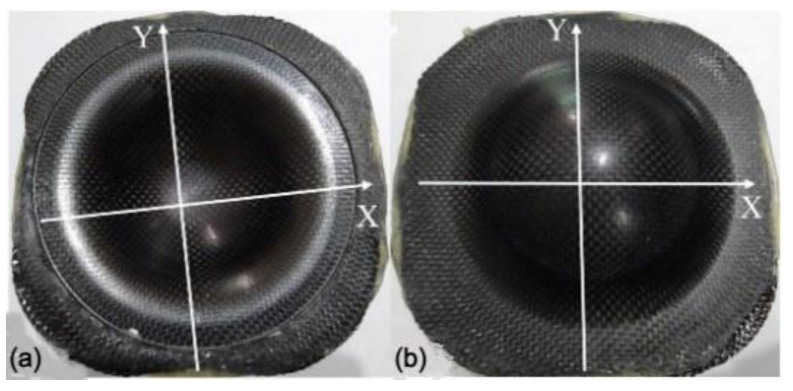
Deep drawing of composite-90 specimen: (**a**) front view, (**b**) back view.

**Figure 5:**
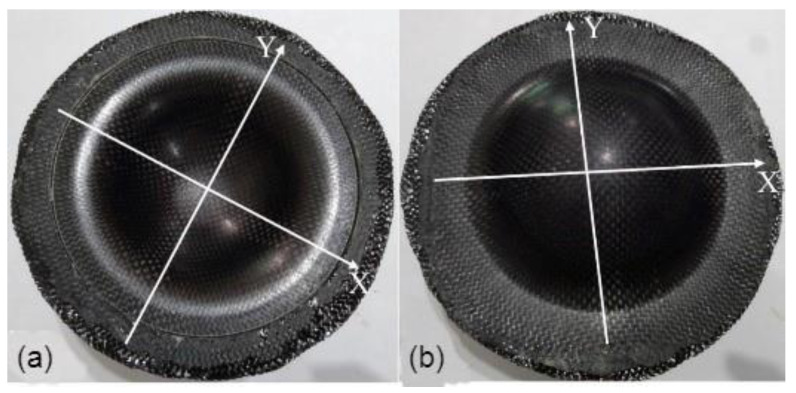
Deep drawing of composite-15 specimen: (**a**) front view, (**b**) back view.

**Figure 6:**
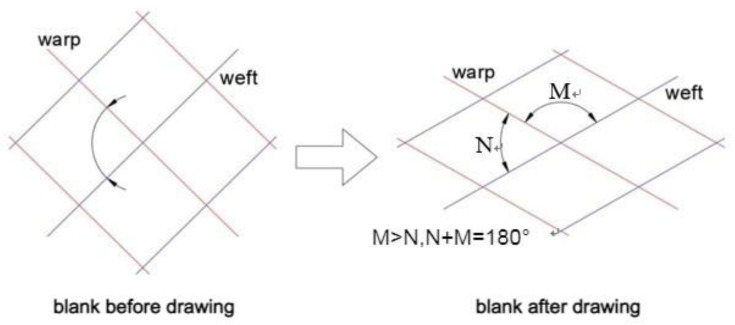
The definition of fiber intersection angle N after drawing and curing.

**Figure 7:**
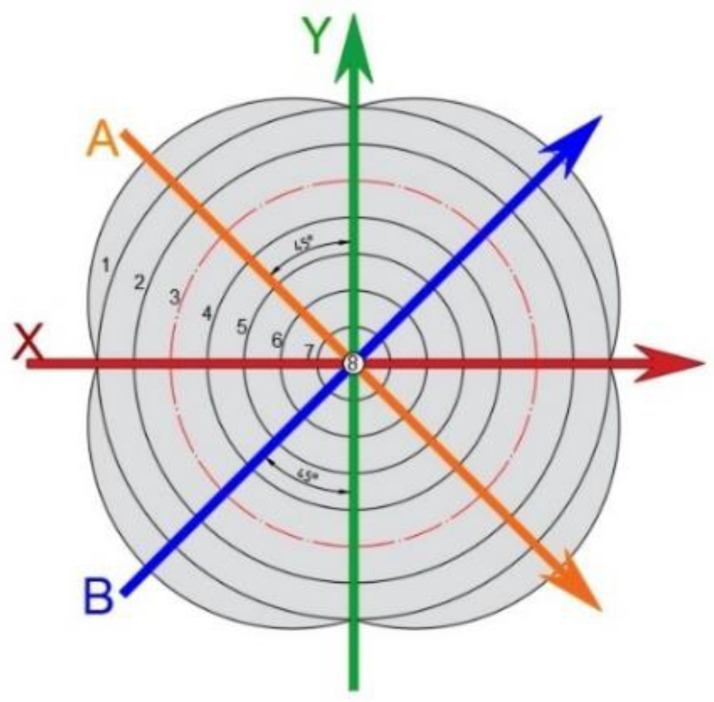
Lines and points to measure fiber intersection angles after drawing.

To investigate the fiber angle change between the weft and the warp after drawing, pure composite laminates were deep drawn and presented in this section. After deep drawing and curing, the front and back views of two specimens, composite-90 and composite-15, were shown in Figure 4 and Figure 5, respectively. The coordinates X and Y denoting the weft and warp are presented in these figures. To discuss the fiber angle change after deep drawing and curing, the included angle between the weft and the warp is defined as the fiber intersection angle. As shown in Figure 6, before drawing and curing, angles N and M are 90 degrees. After drawing and curing, the summation of angles N and M is still equal to 180 degrees, and the smaller angle, N, is defined as the fiber intersection angle in this work. The fiber intersection angle of the top layer after drawing at different points is shown in Table 3 for four types of composite specimens. In this table, lines A and B represent 45-degree lines with respect to the coordinates X and Y as shown in Figure 7. Points 1 to 7 as listed in Table 3 denote the points from the outside to the center with even distribution of 10-mm-gap as shown in Figure 7. Hence, point 8 that is not shown in the table represents the center.

As illustrated in Figure 7 for the composite-90 specimen, lines X and Y, which are the weft and warp, are shorter than lines A and B or other radial lines after drawing. This may explain the deformed shape as shown in Figure 4 for the composite-90 specimen. For the composite-15 specimen, since the fiber distribution is more even than that of the other specimens, the outer contour of this specimen looks just like a circle as shown in Figure 5. Because of symmetry, lines X and Y should have the same deformed behavior, and the same points on lines A and B should have the same fiber intersection angles. As shown in Table 3, the fiber intersection angles at the corresponding points are very close, although they are different. These differences may be caused by friction. From Table 3, the smallest fiber intersection angle occurs around points 3 and 4. These two points locate at the transition zone of the specimen with point 3 at the edge and point 4 located further inside. Around points 3 and 4, the stretch along the radial direction should be the most severe in the whole specimen, and this may explain the smallest fiber intersection angles. In addition, the fiber intersection angles of the points on lines X and Y have no change and remain 90 degrees. Similarly, the center of the specimen has a fiber intersection angle of 90 degrees.

### 3.2. Aluminum-Composite Sandwich Plates

**Figure 8:**
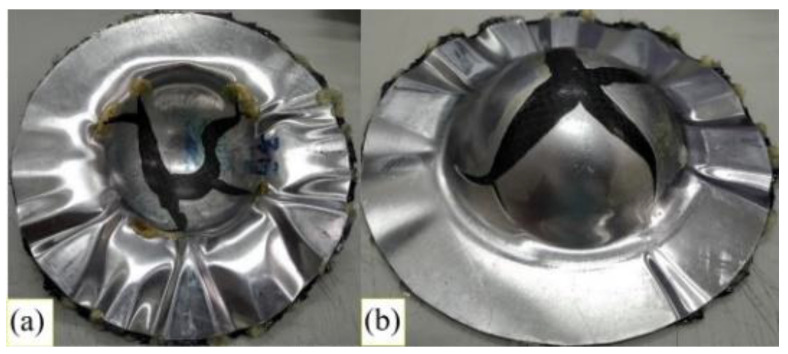
Deep drawing of Al 1050-composite sandwich: (**a**) front view, (**b**) back view.

**Figure 9:**
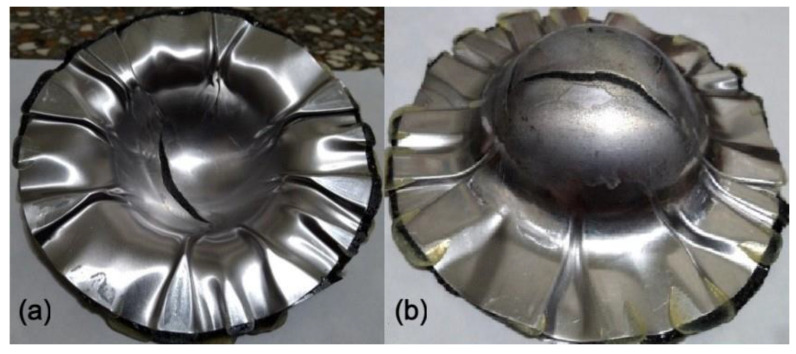
Deep drawing of Al 6061-composite sandwich: (**a**) front view, (**b**) back view.

When aluminum was used as the face layer in the sandwich plate, there were two types of aluminum, 1050 and 6061. The deep drawing of these two types of Al-composite sandwich laminate is shown in Figure 8 and Figure 9. As shown, the fracture condition of the Al 1050 sandwich is much worse than that of the Al 6061 sandwich, and this may be due to the yield and ultimate strengths of Al 1050 being less than those of Al 6061 as shown in Table 1 and Figure 3. According to the condition of fracture and wrinkle in Figure 8 and Figure 9, one may find that it may be difficult to obtain a good quantity workpiece by using these two types of aluminum face sheets.

**Figure 10:**
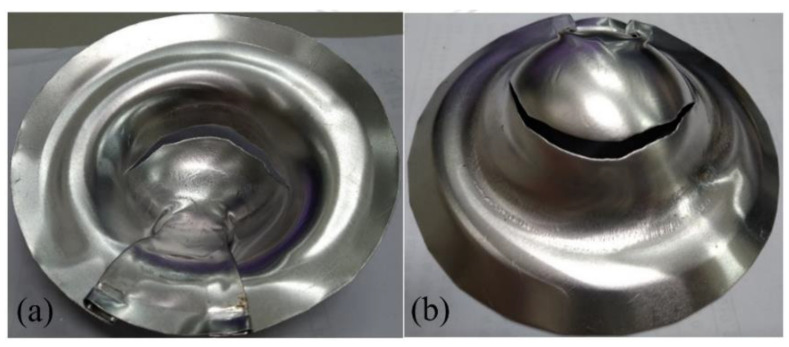
Deep drawing of Al 6061-composite sandwich with 140 °C preheating: (**a**) front view, (**b**) back view.

**Figure 11:**
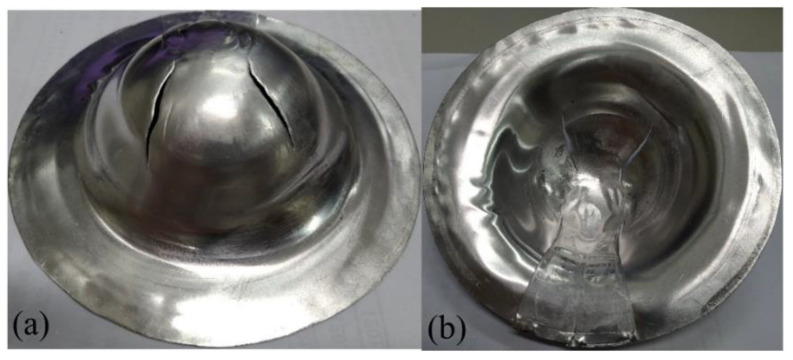
Deep drawing of Al 6061-composite sandwich with 190 °C preheating: (**a**) front view, (**b**) back view.

**Figure 12:**
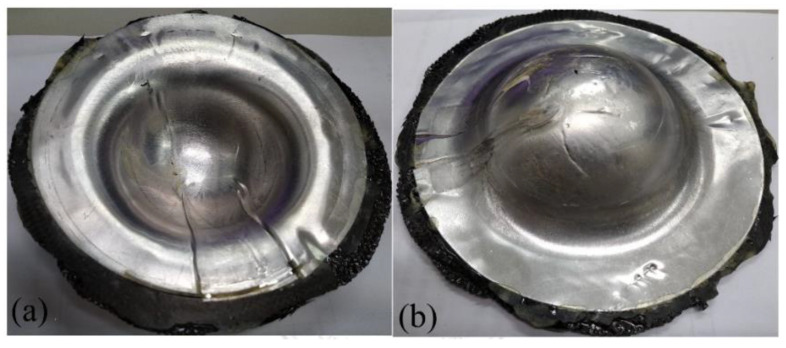
Deep drawing of Al 6061-composite sandwich with 230 °C preheating: (**a**) front view, (**b**) back view.

Moon et al. [6] pointed out that by increasing the die preheating temperature that will affect the blank temperature, the deep drawability of Al 1050 could be promoted because the material became ductile. Therefore, in this work, a two-stage drawing process as described in Section 2 was proposed to investigate the preheat of Al 6061 on the drawability of the Al-composite sandwich plates. The considered preheat temperature was 140, 190, or 230 °C. The deep drawing results are shown in Figure 10, Figure 11 and Figure 12. When compared to the case without preheating as shown in Figure 9, this two-stage drawing process does release the fracture and wrinkle conditions. As the increasing of the preheating temperature, the fracture condition was reduced. The possible reasons may be the better drawability of aluminum, the lower friction between the face and core layers, and the flowability of the resin. As shown in Figure 12, there is no fracture in the face layer. However, wrinkles still appear. These results indicate that it is very difficult to obtain a good quantity Al-composite sandwich structure by deep drawing. Not to mention, the two-stage drawing process makes the manufacturing method very cumbersome.

### 3.3. Steel-Composite Sandwich Plates

**Figure 13:**
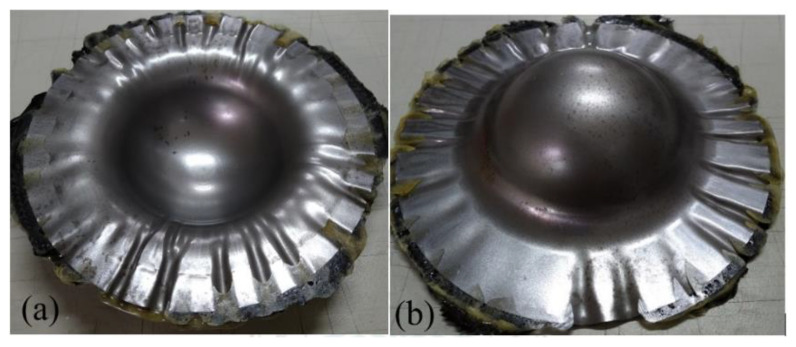
Deep drawing of steel-composite sandwich with blank-holding pressure of 0.6 MPa: (**a**) front view, (**b**) back view.

**Figure 14:**
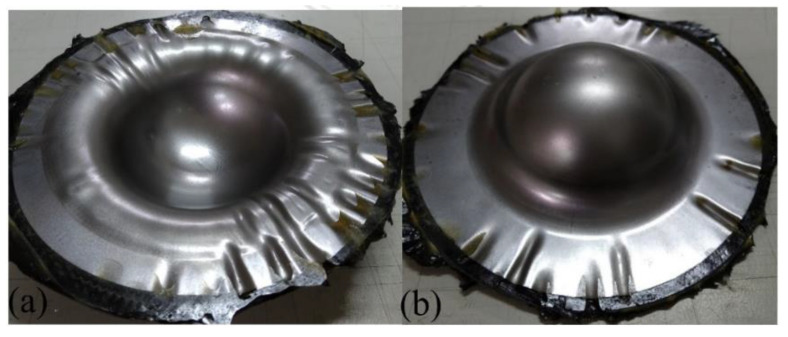
Deep drawing of steel-composite sandwich with blank-holding pressure of 1.1 MPa: (**a**) front view, (**b**) back view.

**Figure 15:**
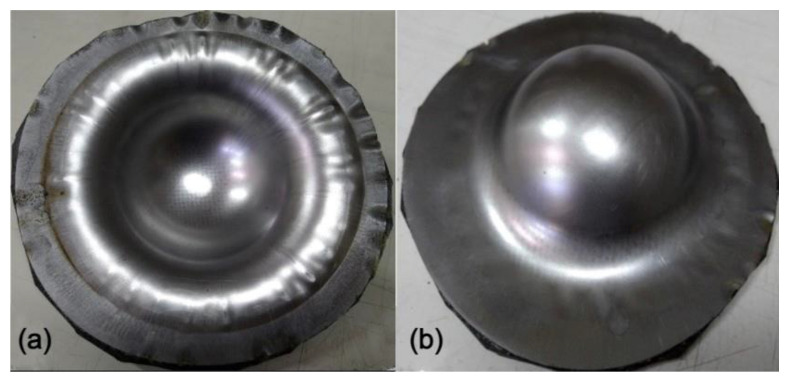
Deep drawing of steel-composite sandwich with blank-holding pressure of 2.5 MPa: (**a**) front view, (**b**) back view.

**Figure 16:**
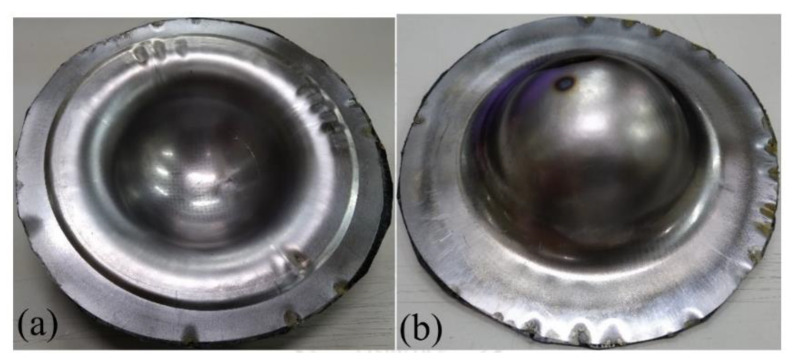
Deep drawing of steel-composite sandwich with blank-holding pressure of 3.5 MPa: (**a**) front view, (**b**) back view.

**Figure 17:**
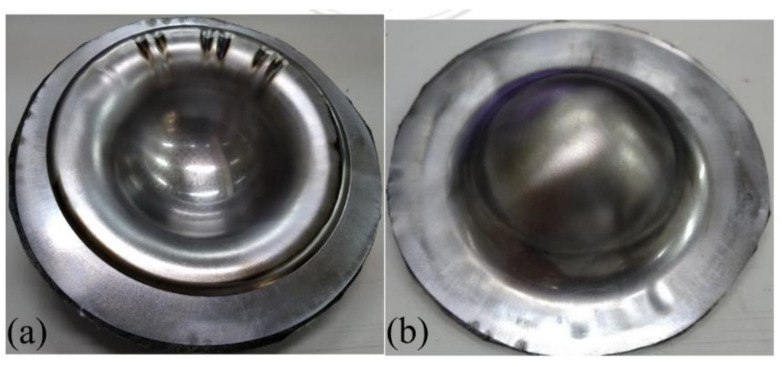
Deep drawing of steel-composite sandwich with blank-holding pressure of 4.0 MPa: (**a**) front view, (**b**) back view.

**Figure 18:**
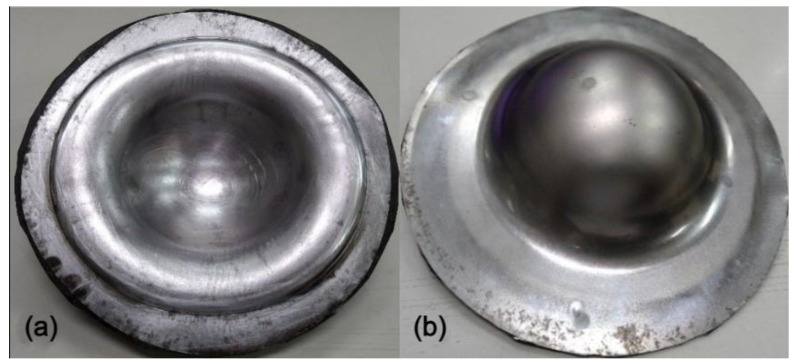
Deep drawing of steel-composite sandwich with blank-holding pressure of 5.5 MPa: (**a**) front view, (**b**) back view.

Generally, steel has better drawability than aluminum as judged from the stress-strain curves as shown in Figure 3. Especially, SPCC steel, a commercial quality cold rolled steel, could be used for forming purposes. This type of steel was combined with the composite materials into sandwich plates and deep drawn under different blank-holder pressure. When the blank-holding pressure was 0.6, 1.1, 2.5, 3.5, 4.0, or 5.5 MPa, the deep drawing results of steel-composite sandwich plates are shown in Figure 13, Figure 14, Figure 15, Figure 16, Figure 17 and Figure 18. From these figures, there is no fracture of the steel face layer. However, wrinkles appear in most cases. The number of wrinkles is counted and listed in Table 4. Three specimens were deep drawn for each case to check the repeatability. As shown, the wrinkle number is slightly varied in the three repeated specimens, which may be due to the variation and non-even distribution of the blank-holder pressure. As shown in this table, the wrinkle number is reduced with the increase of blank-holder pressure. For example, when the blank-holder pressure is 0.6 MPa, there are 19 to 22 wrinkles. When the blank-holder pressure is 5.5 MPa, only one specimen has one wrinkle, and the other two specimens have no wrinkle. Therefore, one could say that a good quality steel-composite sandwich workpiece could be obtained by deep drawing with the blank-holder pressure of 5.5 MPa or above. If the position of the wrinkle is closely examined, as the blank-holder pressure is 2.5 MPa or above, the wrinkle just appears in the outer part of the transition zone and the flange. Hence, if only the hemispherical region is concerned, it may be enough to use the blank-holder pressure of 2.5 MPa or above.

**Figure 19:**
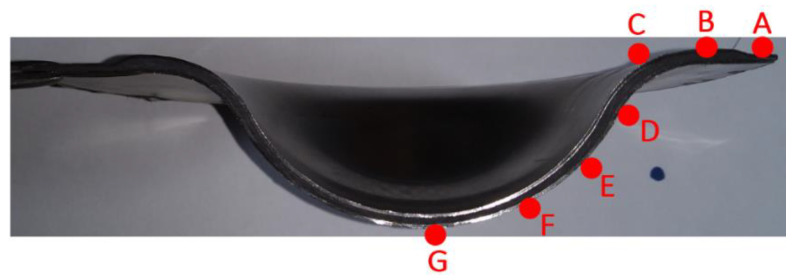
Point labels in the cross-section of steel-composite sandwich for thickness measurement.

In this work, due to the design of the transition zone in the molds, the thickness of the transition zone of the drawn workpiece is concerned. After deep drawing and curing, the workpiece was cut to measure the thickness variation from the flange to the center, and the points named A to G are shown in Figure 19. The thickness of these points is listed in Table 5 when the blank-holder pressure is from 2.5 to 5.5 MPa. The results show that points B, C, and D, which are located in the transition zone, have a larger thickness than points A, E, F, and G, which have almost the same thickness. During the drawing and curing process, points A, E, F, and G are under extension and high surface pressure, while points B, C, and D are under extension and low surface pressure. With the increase of the blank-holder pressure, both the surface pressure and the extension of the former four points are increased and they cause a decrease in the thickness. Since points C and D are mainly under extension, the variation of their thickness with the blank-holder pressure is not evident. However, the thickness of point B increases from 3.1 mm to 4.1 mm with the increase of the blank-holder pressure, while the thickness of point A decreases from 3.0 mm to 2.5 mm. This indicates that the main reason for the thickness increase of point B may be the resin flow from point A to point B during the curing process.

## 4. Conclusions

In this work, the deep drawing behavior of composite laminates and metal-composite sandwich plates is experimentally investigated. In the sandwich plate, the metal face layer could be Al 1050, Al 6061, or SPCC steel. In addition to the original deep drawing process, a two-stage drawing process to improve the drawability is discussed for the Al 6061-composite sandwich plate. For steel-composite sandwich plates, the effect of blank-holder pressure is considered. The results of pure composite laminates showed that after drawing and curing, the weft and warp lines are shorter than the other radial lines, causing the specific deformed shapes for different stacking sequences. In addition, the transition zone at the 45-degree radial lines of the composite laminates shows the most severe reduction of fiber intersection angle after drawing. For Al-composite sandwich plates, fractures and wrinkles are easy to occur on both types of aluminum, 1050 and 6061, by regular drawing. The two-stage drawing process does release the fracture and wrinkle conditions and improves the drawability of the Al 6061-composite sandwich with the increase in preheating temperature. For the deep drawing of steel-composite sandwich plates without preheating, fractures are avoided, and the wrinkle number is reduced as the increase of blank-holder pressure. Hence, a good quality workpiece without fractures and wrinkles could be obtained with the blank-holder pressure of 5.5 MPa or above. If only the hemispherical region is concerned, the blank-holder pressure of 2.5 MPa may be enough to make this region free of fractures and wrinkles.

## Figures and Tables

**Table 1:** Mechanical properties of aluminum, steel, and composite.

Pressure (MPa)	AL 1050	AL 6061	Steel	Composite
Density, g/cm^3^	2.63	2.63	7.83	1.75
Young’s modulus, GPa	63.5	68.9	207	70
Tensile strength, MPa	146	234	382	750
Yield strength, MPa	70	112	202	
Poisson’s ratio	0.33	0.33	0.3	0.07
Ultimate strain	0.1	0.16	0.25	0.01

**Table 2:** Stacking sequences of composite specimens.

Specimen	Stacking Sequence
Composite-90	[(0/90)]_11_
Composite-45	${\left\lfloor \left(0/90\right)\left(\pm 45\right)\left(0/90\right)\left(\pm 45\right)\left(0/90\right)\bar{\left(\pm 45\right)}\right\rfloor }_{S}$
Composite-30	${\left\lfloor \left(0/90\right)\left(30/-60\right)\left(60/-30\right)\left(0/90\right)\left(30/-60\right)\bar{\left(60/-30\right)}\right\rfloor }_{S}$
Composite-15	${\left\lfloor \left(0/90\right)\left(15/-75\right)\left(30/-60\right)\left(\pm 45\right)\left(60/-30\right)\bar{\left(75/-15\right)}\right\rfloor }_{S}$

**Table 3:** Fiber intersection angles at different points of composite specimens after drawing.

**Composite-90**							
**Line**	**1**	**2**	**3**	**4**	**5**	**6**	**7**
A	87.7	82.2	72.4	73.9	78.1	85.8	86.2
B	86.0	84.1	70.3	71.6	79.7	86.1	86.2
**Composite-45**							
**Line**	**1**	**2**	**3**	**4**	**5**	**6**	**7**
A	86.4	80.0	69.9	65.7	72.2	84.3	87.6
B	87.7	82.4	67.8	66.1	74.0	85.9	90.0
**Composite-30**							
**Line**	**1**	**2**	**3**	**4**	**5**	**6**	**7**
A	86.2	77.8	62.1	64.3	75.8	77.7	84.1
B	83.7	78.0	60.3	61.9	76.4	80.1	83.8
**Composite-15**							
**Line**	**1**	**2**	**3**	**4**	**5**	**6**	**7**
A	84.3	76.0	73.8	67.7	69.9	78.1	87.8
B	84.3	76.2	69.7	68.4	74.2	80.1	88.0

**Table 4:** Wrinkle number of steel-composite sandwich under different blank-holder pressure.

Pressure (MPa)	0.6	1.1	2.5	3.5	4.0	5.5
Specimen 1	19	16	9	6	3	0
Specimen 2	22	14	11	5	2	1
Specimen 3	20	15	11	7	3	0

**Table 5:** Thickness on the cross-section of steel-composite sandwich (unit: mm).

Point	A	B	C	D	E	F	G
2.5 MPa	3.0	3.1	3.1	3.0	3.0	3.0	2.9
3.5 MPa	2.8	3.4	3.1	3.0	2.8	2.8	2.8
4.0 MPa	2.6	3.9	3.3	3.1	2.7	2.7	2.6
5.5 MPa	2.5	4.1	3.2	2.9	2.6	2.5	2.5

## Data Availability

Not applicable.
